# Understanding factors that could influence patient acceptability of the use of the PINCER intervention in primary care: A qualitative exploration using the Theoretical Framework of Acceptability

**DOI:** 10.1371/journal.pone.0275633

**Published:** 2022-10-14

**Authors:** Libby Laing, Nde-eshimuni Salema, Mark Jeffries, Azwa Shamsuddin, Aziz Sheikh, Antony Chuter, Justin Waring, Anthony Avery, Richard N. Keers

**Affiliations:** 1 Lifespan and Population Health, School of Medicine, University of Nottingham, Nottingham, United Kingdom; 2 Centre for Pharmacoepidemiology and Drug Safety, Division of Pharmacy and Optometry, School of Health Sciences, University of Manchester, Manchester, United Kingdom; 3 NIHR Greater Manchester Primary Care Patient Safety Translational Research Centre, Manchester Academic Health Sciences Centre (MAHSC), University of Manchester, Manchester, United Kingdom; 4 Faculty of Health Sciences, University of Hull, Hull, United Kingdom; 5 Usher Institute, Edinburgh Medical School, University of Edinburgh, Edinburgh, United Kingdom; 6 Haywards Heath, West Sussex, United Kingdom; 7 School of Social Policy, Health Services Management Centre, University of Birmingham, Birmingham, United Kingdom; Flinders University, AUSTRALIA

## Abstract

**Introduction:**

Medication errors are an important cause of morbidity and mortality. The **p**harmacist-led **I**T-based i**n**tervention to reduce **c**linically important medication **er**rors (PINCER) intervention was shown to reduce medication errors when tested in a cluster randomised controlled trial and when implemented across one region of England. Now that it has been rolled out nationally, and to enhance findings from evaluations with staff and stakeholders, this paper is the first to report patients’ perceived acceptability on the use of PINCER in primary care and proposes suggestions on how delivery of PINCER related care could be delivered in a way that is acceptable and not unnecessarily burdensome.

**Methods:**

A total of 46 participants living with long-term health conditions who had experience of medication reviews and/or monitoring were recruited through patient participant groups and social media. Semi-structured, qualitative interviews and focus groups were conducted face-to-face or via telephone. A thematic analysis was conducted and findings mapped to the constructs of the Theoretical Framework of Acceptability (TFA).

**Results:**

Two themes were identified and interpreted within the most relevant TFA construct: Perceptions on the purpose and components of PINCER (Affective Attitude and Intervention Coherence) and Perceived patient implications (Burden and Self-efficacy). Overall perceptions on PINCER were positive with participants showing good understanding of the components. Access to medication reviews, which PINCER related care can involve, was reported to be limited and a lack of consistency in practitioners delivering reviews was considered challenging, as was lack of communication between primary care and other health-care providers. Patients thought it would be helpful if medication reviews and prescription renewal times were synchronised. Remote medication review consultations were more convenient for some but viewed as a barrier to communication by others. It was acknowledged that some patients may be more resistant to change and more willing to accept changes initiated by general practitioners.

**Conclusions:**

Participants found the concept of PINCER acceptable; however, acceptability could be improved if awareness on the role of primary care pharmacists is raised and patient-pharmacist relationships enhanced. Being transparent with communication and delivering streamlined and consistent but flexible PINCER related care is recommended.

## Introduction

Medication errors are an important cause of morbidity and mortality [1, 2]. The National Health Service (NHS) has reported that in England, approximately 237 million medication errors are made annually [3, 4], with around 66 million having the potential to cause harm [5]. Seventy-one percent of this 66 million can be attributed to primary care [3, 4], an area in which patients are normally expected to self-administer their medicines and are generally responsible for organising their medication monitoring appointments [6]. In terms of adverse outcomes, approximately 4% of hospital admissions are linked to drug-related morbidity [7, 8], with avoidable drug reactions contributing to 1700 and resulting in 700 deaths per year, costing the English NHS £98.5 million per year [5].

In the developed world, novel computer technologies have been utilised to improve medication safety across the length of the medication pathway (i.e. from clinical decision-making and prescription through to dispensing and adherence) [9–13]. With a particular focus on the primary care setting, one prominent evidence-based prescribing safety and medication monitoring intervention is the **p**harmacist-led **I**T-based i**n**tervention to reduce **c**linically important medication **er**rors (PINCER) [14, 15]. The PINCER tool is used to systematically search General Practice electronic clinical record systems using a set of 13 statements of potentially hazardous prescribing events, known as prescribing safety indicators, to identify patients that are potentially at risk of harm from their medicines. The indicators have been designed specifically to identify hazardous prescribing associated with certain long term medical conditions and medicines that require regular monitoring via blood tests (listed in Table 1) [16].

**Table 1:** Medical conditions and drugs associated with PINCER prescribing indicators [16].

**Associated medical conditions**
Gastrointestinal bleeding
Asthma
Stroke
Heart failure
Acute kidney injury
**Drugs that require regular monitoring (i.e. blood tests required)**
Angiotensin converting enzyme (ACE) inhibitor
Long-term loop diuretic
Methotrexate
Lithium
Amiodarone

General practice pharmacists trained in education outreach communicate the results of the searches with General Practitioners (GPs) and devise a collaborative action plan to reduce future medication related risk [16–18].

PINCER has been tested and shown to be effective in a cluster randomised controlled trial [14], has been successfully implemented on a regional scale and rolled out across the English NHS 2018–21 [16]. Process evaluations of PINCER thus far have explored stakeholder and staff perspectives on the intervention, contextual factors that could influence its use and effectiveness [16, 19, 20] and potential cost-effectiveness [21]. More recent work, including an unpublished evaluation with staff and stakeholders, has also proposed strategies that could be used to support optimal and sustainable use of PINCER across different primary care settings [22] however, there has been limited work done to explore patients’ opinions on the use of PINCER in primary care.

Patient acceptability is an important aspect in the success of medication safety interventions [23] and can influence patients’ adherence to treatment and clinical outcomes relating to the intervention [23]. Recommendations for clinical treatment guidelines tend to prioritise achieving optimal clinical outcomes and although they may consider the acceptability for patients, patient experience and how new practices could impose on patients is often overlooked [24]. NICE medicine optimisation guidelines [25], the response by the Short Life to the World Health Organisation’s (WHO’s) 2017 Medication Without Harm campaign [26] and the 2019/20 Quality and Outcomes Framework (QOF) describe the components of PINCER, outline its effectiveness and advocate its use, however, there are no published recommendations, as yet, on how PINCER related care could be delivered optimally for patients.

Informed by the Theoretical Framework of Acceptability (TFA) [23], this paper reports the first study to investigate patients’ views on the use of PINCER in general practice and generate suggestions on how PINCER related care could be delivered to patients in a way that is acceptable, does not pose any unnecessary burden and fosters patient engagement.

## Methods

### The Theoretical Framework of Acceptability

The TFA facilitates evaluation of intervention acceptability based on lived or perceived experiences of those delivering or recipients of an intervention [23, 27]. As such, it goes further than focusing on intervention components alone and can account for personal, organisational and other contextual factors that could also influence both intervention delivery and acceptance [23, 27]. The TFA consists of seven constructs (shown in Table 2), through which acceptability can be assessed prior to, during and post intervention delivery [23].

**Table 2:** Constructs and descriptions of the Theoretical Framework of Acceptability.

Construct	Description
Affective Attitude	How an individual feels about the intervention
Burden	The perceived amount of effort that is required to participate in the intervention
Ethicality	The extent to which the intervention has a good fit with an individual’s value system
Intervention Coherence	The extent to which the participant understands the intervention and how it works
Opportunity Costs	The extent to which benefits, profits or values must be given up to engage in the intervention
Perceived Effectiveness	The extent to which the intervention is perceived as likely to achieve its purpose
Self-efficacy	The participant’s confidence that they can perform the behaviour(s) required to participate in the intervention

Information taken from page 8 of 13, Sekhon, Cartwright & Francis (2017) [23]

The TFA has been used successfully to assess recipients’ acceptability of other relevant interventions including disease prevention approaches [27], chronic disease management in primary care [28] and pharmacist-facilitated medication reviews [29]. In this current study, the TFA was considered the most appropriate to identify factors that could influence patient acceptability of the use of PINCER in primary care and help interpret patients’ perceived or lived experiences of PINCER related care.

### Study design

This study, which was part of a larger qualitative evaluation of a prescribing safety clinical support (CDS) system and PINCER during the national roll-out phase, focused on patient acceptability of the use of the PINCER intervention in primary care, England. It was conducted using semi-structured, individual and group interviews and focus groups.

### Sampling and participants

Recruitment targeted patients aged 18 years or over who were able to provide written informed consent. The initial recruitment strategy included asking health care professionals to identify and approach eligible patients, who had been identified in PINCER searches, to take part in the study. However, due to winter pressures and the impact of the pandemic on health care professionals’ workload and ability to engage in research activities, we were unable to pursue this option. Therefore, participants who were patient members of a patient participation group (PPG) and/or were living with a long-term health condition were sought (a PPG normally consists of patients, carers and practice staff who regularly meet to discuss issues and identify potential ways in which service provision and patient experience can be enhanced within the practice they are registered at or work in [30]). In order to meet recruitment targets and ensure there was a wider spread of demographics amongst participants, a targeted advertisement was placed on Facebook and ran for six weeks in order to recruit other participants who met the eligibility criteria. This was facilitated and managed by an external company (https://www.healthresearch.study) who specialise in recruiting patients, for research purposes, using social media. The advertisement had a link to a short screening questionnaire which ran on Jisc online surveys software (https://www.onlinesurveys.ac.uk/). To ensure participants had experience of aspects of care relevant to PINCER, it was a requirement that they had recent experience of a medication review at their general practice, which being identified through PINCER can result in, and/or were on medication that required regular monitoring.

Recruitment covered four regions of England: East Midlands, Wessex, South Midlands & Thames Valley and Yorkshire & Humber where PINCER had been implemented early in the national rollout phase. PPGs were approached by the National Institute for Health and Care Research (NIHR) Clinical Research Network (CRN), a body set up to help coordinate and support research activities, who informed the research team of any expressions of interest. All eligible PPG and social media recruited participants who agreed to take part were provided with an information sheet and had the opportunity to ask questions prior to providing informed written consent.

### Data collection

At the start of the interviews and focus groups, as participants were likely to have limited prior knowledge of PINCER, an overview of the PINCER intervention was provided to them in lay terms. It was also explained to participants that the research team were unaware if their practice were currently or had previously used PINCER or if they had been a direct recipient of PINCER related changes to care. A semi-structured interview template was used (S1 Appendix) which was designed to generate discussions surrounding the use of PINCER in primary care, participants’ experiences of medication reviews and changes and/or medication monitoring and elicit opinions on these aspects. LL, who has a previous background in nursing and has experience in qualitative health-care research, conducted the interviews and facilitated the focus groups with one other researcher (NS, a senior research fellow and pharmacist or MJ, a researcher with extensive experience and expertise in qualitative research interviewing).

All focus groups and interviews were conducted between December 2019 and December 2020, were audio-recorded and transcribed verbatim by a University approved transcription service. Identifiable data were removed during the transcription process and returned securely on a separate document for each individual audio-recording/transcript.

### Data analysis

An initial inductive thematic approach was taken which was guided by the six phases of qualitative analysis described by Braun and Clark [31]. All audio-recordings were re-played and crosschecked with the transcripts in order to correct errors and to re-familiarise LL with the data set at the start of the analysis. QSR NVivo 12 Pro was used to organise the data, code the transcripts and identify emerging themes. An iterative process was used by LL to review and define the themes and sub-themes and produce a coding framework. MJ then coded nine of the 27 transcripts independently and results were compared with LL’s which facilitated reflection on interpretation and consistency of coding. The coding framework was then discussed and finalised with the wider research team (MJ, RK and AC—our patient and public representative) which helped ensure the themes identified and coding framework were a good representation of the data [32, 33]. An abductive approach, as described by Tavory & Timmermans [34], was then taken in which the themes and sub-themes were mapped to the seven constructs of the TFA [23]. LL discussed the results of this final stage of the analysis with RK and MJ and made some refinements based on these discussions.

### Ethical approval

Ethical approval was granted by the East Midlands–Nottingham 2 Research Ethics Committee and the NHS Health Research Authority (IRAS ID: 212446). This covered recruitment in England, UK.

## Results

A total of 46 participants took part in an interview or focus group, this included 26 PPG members from five PPGs and 20 recruited from social media. Of these, 29 were female, 17 were male. The age range, for those who disclosed it (n = 43), was 20–82 years (mean = 62 years). Two focus groups, one with eight participants and one with seven, two group interviews, each with three participants and 25 individual interviews were conducted. The focus groups, one individual interview and two group interviews were conducted face-to-face with PPG participants at their general practice. The remaining four PPG participants and all 20 social media recruited participants were interviewed individually over the telephone due to practicalities and in compliance with COVID-19 guidelines at time of interview. The focus groups duration ranged from 48–59 minutes (mean = 54 minutes), group interviews ranged from 33–45 minutes (mean = 39 minutes) and individual interviews ranged from 12–41 minutes (mean = 22 minutes). Participants were offered a £20 high street voucher to thank them for their time.

Two main overarching themes were identified which mapped to four TFA [23] constructs: Perceptions on the purpose and components of PINCER (Affective Attitude and Intervention Coherence) and Perceived patient implications (Burden and Self-efficacy). The main themes and how they mapped to the TFA constructs are outlined in Table 3 and described in more detail in the appropriate section. The prefix given with the participant identifier codes denotes whether they had been recruited from a patient participation group (PPG) or via social media (SM).

**Table 3:** Summary of results from mapping the themes to the constructs of the Theoretical Framework of Acceptability [23].

Perceptions on the purpose and components of PINCER	
Affective Attitude	It was proposed that using an IT-based system such as PINCER could help with the general capacity of the NHS
	The potential improvements to the service offered by practices using PINCER were identified, particularly by participants who had experienced medication errors and those who offered reasons as to why they could occur
	Pharmacists were viewed as being well placed to identify potential causes of errors that patients may not have awareness or understanding of as well as communicate issues surrounding medication safety effectively with patients
	Awareness of, and opinions on, the role and expertise of pharmacists appeared to influence the willingness to accept or engage in changes that were initiated by a pharmacist. It was acknowledged that some patients are more resistant to change and may be more hesitant to consult with or accept recommendations or advice from health care practitioners other than doctors
	A perceived benefit of using PINCER was that it could allow GPs to have more contact time with patients and allow health care practitioners to focus more on their areas of expertise more generally
	Using PINCER was thought of as beneficial in being able to improve patient safety providing the communication was effective and the patient was given a central role
Intervention Coherence	The need for adequate training for pharmacists and other health care practitioners who would be using PINCER was recognised
	There were some concerns surrounding the frequency of running the searches and how consistent the service offered to patients, based on the use of PINCER, would be
	There was some scepticism around PINCER being a cost-cutting exercise
	It was identified that PINCER would not be able to monitor levels of adherence to medication
	Pharmacists offering patients clear instructions on how to take their medication and explaining potential side effects in a way they could understand was suggested as a possible way to avoid errors and encourage adherence (i.e. as an added feature that could enhance the intervention)
	Having systems that did not communicate with each other between primary care, secondary care and community pharmacies was seen as being problematic (i.e. could impact on effectiveness)
**Perceived patient implications**	
Burden	Offering a medication review to patients who had been identified in the PINCER searches was considered worthwhile even if no changes were made during the review
	Although patients appreciated having medication reviews and found them useful, there was experience of limitation in the availability of these appointments
	Consistency of the practitioner conducting a medication review was an important consideration in terms of how patients were able to engage in and get benefit from the review
	Using different modes of delivery for medication reviews was perceived to open up more opportunity for patients to participate in a review with some finding remote consultations more convenient and others reporting that face-to-face appointments facilitated better communication
	Having different medications reviewed at different times was also considered problematic and an issue that some participants, who had experienced this, tried to resolve themselves
	An issue highlighted was that when changes were made to one or some patients’ medicines, it can result in the patient having to make additional prescription collection trips due to the changes causing a lack of synchronisation in start dates with other medications
	There were reports of reliance on patients to record and understand changes that were made to their medications during remote reviews which was felt to be challenging
	It was advocated for those considered most vulnerable that members of their social network should be able to accompany them to medication reviews and that systems should be in place to facilitate optimal communication between health care practitioners and this group of patients
Self-efficacy	Although it was advocated that patient involvement is important and central to the medication review process, it was also suggested that that health care practitioners should initiate appropriate conversations for patients who may be more reluctant or who feel less able to question their medications or treatment regimen

### Perceptions on the purpose and components of PINCER

#### Affective attitude

Views on the purpose and components of PINCER were mainly positive, giving an indication of acceptability on these particular aspects of the intervention. It was proposed that using IT-based system could help with the general capacity and efficiency of the NHS, especially at a time when demand is on the increase. The following response was given pre-pandemic, prior to the workload of the NHS being impacted upon by the COVID-19 response, indicating its added significance given current challenges of the workload burden posed by the pandemic recovery phase [35].

*"Yes*, *as I say if you think about it the health service … the population is going to be ever growing so the health service is going to actually have to change its ways so it has got to be more efficient but more efficient with computers…*.*"* **(PPG, Par 1)**

The potential improvements to the service offered by primary care establishments using PINCER were recognised and appreciated, particularly amongst participants who had experienced medication errors and those who offered reasons as to why errors could occur. For example, the following response was given by a participant when discussing an error a relative had experienced.

“*I mean as soon as a district nurse had visited … and flagged it up urgently with the doctor [his] medication was changed very*, *very rapidly and he recovered quite well … but it was … scary at the time … So*, *if there is something … if there is some way that that is monitored either electronically or whatever doing those medication searches*, *that highlights that something is … I wouldn’t say missed but overlooked*, *then I am more in favour of that*.*”* **(SM, Par 16)**

The following quotation from another participant illustrates the point when they realised that a complication they had experienced could possibly have been avoided through the use of PINCER.

*“I was on a drug without being on a stomach protector and I ended up with an ulcer … Yes*, *so that [PINCER] would have actually probably prevented that happening*. *So*, *it sounds excellent*.*"* **(SM, Par 17)**

Understanding and opinions on the role and expertise of pharmacists influenced acceptability surrounding PINCER. Most participants viewed pharmacists as being the most appropriate health care practitioners to lead on PINCER and best placed to identify wider issues surrounding potential medication errors.

*"Personally*, *I think that is better like … not in any offence to GPs and stuff like that but pharmacists are the ones that are specifically trained in medication*, *so they are going to be the best people to look at it*.*"* **(SM, Par 13)**

This view, however, was not held by all with some questioning aspects surrounding the comprehensiveness of interactions between pharmacists and GPs as well as pharmacists’ motives for suggesting or making changes to medication. Furthermore, in terms of receptivity to PINCER related care, there was some indication that patients may be less willing to accept suggested changes to care that had been initiated by a pharmacist rather than a GP.

*"I don’t mind the pharmacist having some input*, *if there is a new alternative but I don’t think they should initiate it*.*"* **(SM, Par 4)**

The quotations above also allude to issues of trust in health care professionals. Some participants perceived pharmacists as being trustworthy, and although they commented that their perception was that the amount of training pharmacist undertake was shorter in duration than that of doctors, it was considered adequate for the purposes of running PINCER. For example, “*I do trust my pharmacists*, *they do have years of training*, *not as much as a doctor of course but they can help you with minor ailments and they know about the names of drugs and what the drugs do more than anything else so I am quite happy with that*.*”* **(PPG, Par 4)**

Levels of trust in pharmacists also seemed to be influenced by experiences of interactions with them. The following response was given by a participant who had undertaken some background reading on PINCER prior to being interviewed, on the basis of the study information they had been sent.

*“Previously I haven’t had a lot to do with pharmacists and when I heard about this intervention [PINCER] my immediate thoughts were I wouldn’t trust them as much as I would trust the doctor but since … I have had all of these changes to my medication*, *I have had a lot more to do with the pharmacist and she has been really good advising me and you know when to take medication for instance …”* **(PPG GI 2, par 3)**

Participants also acknowledged that some misunderstandings on the role of general practice pharmacists exist, including a lack of awareness and inability to differentiate between the remit of general practice and community-based pharmacists.

*"… you know somebody could think oh*, *that is just the woman that hands you the prescription over the counter at the chemist when actually it is somebody who has done loads of training and is very experienced and I think there is a lot of … a lack of knowledge about who pharmacists are and what they do …”* **(SM, Par 8)**

In order to resolve these misunderstandings, it was proposed by a few participants that awareness surrounding the role and expertise of general practice based pharmacists should be raised. In doing so, it was suggested that the credibility, acceptance and trust in pharmacist based decisions could be enhanced.

*"… they [pharmacists] are the experts in that field and I don’t think we necessarily give that credit and understanding so within this I think we need to make sure that we promote the understanding of what a pharmacist can do so that people don’t think they have got to automatically jump back to the GP*.*"* **(PPG Focus Group 1, Par 9)**

A perceived positive consequence of PINCER being pharmacist-led was that it would potentially lesson GP workload which would increase GP contact time for patients and enable health care practitioners to focus on their areas of expertise more generally.

*"I think anything that frees up doctors’ clinical time to see patients about actual you know things that are wrong rather than reviewing the drugs then it is a benefit of everybody*. *If it frees up doctor time*.*"* **(PPG Focus Group 1, Par 3)**

It was also recognised that by using PINCER, health care practitioners could improve patient safety and thereby meet the purpose it was intended for. However, this improvement was felt to be dependent on communication surrounding PINCER related care involving the patient and being transparent in nature.

*"… I don’t think I would have a problem with that [changes being made on the basis of PINCER] providing obviously that it is discussed neutrally with the GP and the patient*. *I think it does need all three parties to have some kind of input*.*"* **(SM, Par 12)**

Transparency in communication included explaining that reasons for any proposed changes were evidence-based and to reassure patients that decisions behind the changes were in their best interests.

*"… well for me personally if somebody came to me and said look we’re going to change your medication*, *and this is why we’re doing it*, *and these are the benefits … I would be quite happy to listen to the practitioners and go with their advice*…*"* **(SM, Par 14)**

Without this type of open communication, it was felt that patients might get suspicious of the reasons behind suggested changes and be less likely to consider or adhere to them.

### Intervention Coherence

Although there were some disparities in levels of understanding surrounding the role, scope of practice and knowledge base of primary care pharmacists, one aspect many participants shared was the recognition for the need of intervention specific training for pharmacists and any other health care professional involved with PINCER. The following quotations came from discussions generated in focus groups.

*“I think [using PINCER would be beneficial] so long as it is put in with the correct training and a clear understanding of how it should be used …”* **(PPG Focus Group 1, Par 9)** and *“… with the appropriate education I think PINCER will have a very positive effect on practices and therefore on patients*” **(PPG Focus Group 1, Par 6)**

As well as seeing the benefits of training, the need to have up to date training was also identified in order to maintain proficiency amongst users, for example, *“… there needs to be regular reviews on training and especially at times when that software is changed or amended*.*”* **(PPG Focus Group 1, Participant 8)**.

Although it was explained that PINCER is an audit tool and as such, the searches are designed to be run every six months, concerns were raised surrounding how effective or reliable this would be. Responses in relation to this highlighted there was some consensus in preferences for the frequency of running the searches, with these preferences being more in line with the functionalities of clinical decision support systems which produce alerts at the point of prescribing. Questions surrounding safety were raised in regards to the six monthly search cycle.

*"I think it [PINCER] should be used but I am concerned that it is only running every six months*, *if I run my virus check once every six months I would have no money in my bank account … It [PINCER] should be running in the background all of the time*. *You know and when a health care professional looks in to your medical history or whatever … It should be there and it should be running and if they put something in … it should say oh hang on a minute*…*"* **(PPG Focus Group 1, Par 2)**

The participants’ level of understanding of the intervention was also highlighted through the identification and discussion of aspects that PINCER was not designed to do or address. For example, there was some scepticism and misunderstanding surrounding it being a cost-cutting tool … *“…the bottom line is always cost isn’t it…”* **(PPG GI 2, Par 3)**, which although it may be cost-effective, it was not designed to cut prescribing related costs for practices. Better understanding, however, was demonstrated through responses relating to aspects which can be problematic, for example, adherence that PINCER is neither able nor designed to address.

*"That is the other thing that PINCER doesn’t take into account … that your medication review works on what you’re prescribed not what you take …”* **(PPG GI 1, Par 1)**

This understanding also extended to participants’ identifying features that could enhance outcomes relating to PINCER as well as factors that could impact on its effectiveness. For instance, recommendations made in order to help patients understand more about their medication and potentially avoid errors and/or encourage adherence included pharmacists or other health care practitioners giving clear instructions to patients on how to take their medication and explain the risk factors in a way that they would understand.

*"I quite understand why any medication comes with a piece of paper with risk factors but I think a lot of people don’t understand those risk factors*. *Now I don’t know whether it would be the remit of the pharmacist to explain to people [who] are certainly on multiple medications*, *to actually tell them what the risk factors mean because although you try to get it across to people*, *when you say one in hundred*, *one in a thousand*, *one in ten … what does that mean*?*"* **(PPG GI 1, Par 2)**

As they have separate record systems which do not automatically share information, the unlikelihood of medicines prescribed by secondary care or purchased over the counter being picked up by PINCER was also mentioned by a few of the participants.

*"I am just on one [primary care] prescribed medicine*… *lansoprazole … but … once a year now I have to have something called zoledronic acid which is for Osteoporosis … because [it is] not actually prescribed by the GP it is the hospital that gives that through an intravenous infusion so I don’t know how the computer would be able to like work one with the other*?*"* **(PPG, Par 1)**

This lack of shared information between systems was thought to place extra responsibility on patients to ensure their primary care practitioners were aware of any non-primary care prescribed medicines they were taking. Not only was this considered burdensome, it was also identified as an area in which omissions or mistakes could easily happen.

*“Yes*, *because you see when you go and see a consultant [in secondary care] you actually have to say to them about what medications you’re on and then you have to try and remember*, *even if you go and see a consultant*.*”* **(PPG, Par 3)**

### Perceived patient implications

#### Burden

The main perceived source of burden and effort required from patients related to medication review consultations and prescription changes, which patients who are identified as being at potential risk of harm from their medicines though the PINCER searches are likely to experience.

Most participants advocated that medication review consultations were worthwhile if conducted well, indicating that being a recipient of them, in itself, was not considered burdensome. Furthermore, offering a medication review to patients who had been identified in the PINCER searches was considered beneficial even if no changes were made during the review.

*"… if you get a proper [medication] review … with the professional in situ and you have been flagged up [by PINCER] because something has occurred*, *now it maybe that you walk out of that door with exactly what you walked in with*, *having reconsidered it all*, *found out how they are all working … or it maybe that you come out with something better to try or something different to try or some other advice … that might make a difference*.*"* **(PPG Focus Group 1, Par 9)**

However, although experienced or perceived positive experiences of medication reviews were reported, negative aspects were also highlighted. This included consistency of seeing the same practitioner which could impact on how patients were able to engage in and get benefit from a PINCER related medication review. Seeing different practitioners for reviews required additional effort from patients.

*"… Yes*, *it [seeing the same practitioner] makes it easy … you feel like oh*, *this person understands what I am telling them because they are the ones who prescribed me this medication … and they made notes on the system but when the next GP sees the notes on the system*, *they might not understand everything like you know … when the previous GP listened to my issue … and sometimes they might have missed out something or I might miss out something as well when I am explaining again*.*"* **(SM, Par 3)**

Negative experiences of medication reviews also seemed to contribute towards a decline in patients’ willingness to book or attend them. The quotation below gives an example of a participant not feeling listened to and who perceived the review as more of a ‘tick box’ exercise which was also how others described their experiences.

*“… in recent times it [medication review] has literally been have you got any problems*? *No not really*, *OK*. *It has been as yes as superficial as that and to be brutally honest I have given up talking about my perceived side effects of the medication*, *because I have never had any joy out of raising them so eventually I just gave up*.*”* **(SM, Par 1)**

This goes against views that, in order to work well, PINCER related care and any recommended changes to be made require that there *“… has got to be some conversation…”* **(PPG, Focus Group 1, Par 9)** between the patients and healthcare professionals and that *“…they [the patients] still feel that they are at the centre of this…”* **(SM, Par 8)**

There was a shared feeling that general practice did not have much capacity to offer the type of medication review that may need to be conducted based on the findings of PINCER searches with accessibility issues being raised.

*"some of us are lucky enough to get*, *you know if you do have an annual review it is great because you can raise those things*, *or you can raise them with your GP if you get chance in an appointment but they [review appointments] are a bit like gold dust*…*"* **(PPG Focus Group 1, Par 9)**

The quotation below was from a participant who was advised via the online ordering system that they needed to speak with a health care professional before some items on their prescription could be re-issued.

*“I telephoned the surgery*, *it took a couple of days to be able to get through on the telephone*, *it is very busy*, *just engaged the whole time*, *and I had to wait*, *I think it was eight days to be able to speak to*, *I think it was the doctor I spoke to in the end who just said yes … keep taking the medication*, *it is still OK for you*, *that is fine…”* **(SM, Par 17)**

Using different modes of delivery, such as conducting reviews via telephone, was thought to open up more opportunities in terms of being able to access and engage in a medication review. However, some participants perceived not being able to attend face-to-face consultations as being a barrier to effective communication. Having a video consultation, which was not something the participants in this study had experienced, was a suggested method that could help overcome this barrier. The following suggestion was offered pre-pandemic, before any social distancing measures were known of or were in place.

*“So sometimes a lot of the consultation isn’t just about what you’re saying is it*? *I know you can pick up tones over the phone but you can’t see how a person is responding*, *the visual cues or … so sometimes FaceTime might work for certain people because when you’re just talking to somebody on the phone you can’t always get across how they are feeling about something*.*”* **(PPG Focus Group 2, Par 3)**

Another issue highlighted that related to both medication reviews and prescription changes was lack of synchronisation which could make managing these things difficult for patients. For instance, having different medications reviewed at different times seemed problematic and was an issue that some participants had tried to resolve themselves. The following quotation mentions medicines that relate to the PINCER indicators.

*"… it’s very difficult if you are on multiple medication*, *to get them all reviewed at the same time*. *I mean I am on six items*, *if anything gets changed it all goes out of sync and I have tried many times … can you put it for all … because it is silly … doing your lansoprazole in January*, *your naproxen in June*, *that is something that I think needs tightening up*. *… They are talking about trying to save patients convenience and surgery time*, *and that really is a waste of time [to] keep having to go and make an appointment for a review*.*"* **(PPG Group Interview 1, Par 2)**

Similarly, in relation to prescriptions, when changes were made to one or some of the medicines on an existing repeat prescription, participants commented that this normally meant start dates to the changed or additional medicines become misaligned with start dates of other items on the prescription. This misalignment results in additional prescription collection times for patients, which was considered particularly problematic for those less able to travel, especially during lockdown when access to public transport and entry to indoor spaces was limited and posed an increased risk of exposure to COVID-19.

*"*… *I had already had this*… *medication for nearly 20 years … then another medication is popped on … They are two weeks out of sync so now I am going to the pharmacists four times a month instead of twice a month and all right I am relatively young and healthy*… *but*… *I know that many people who have long term medication conditions*, *that would be a huge imposition you know talking about bus journeys … So actually*, *if you’re going to prescribe some completely new drugs*, *try and make them coincide with the existing drug dates*, *prescription dates*.*"* **(SM, Par 1)**

There were also reports of reliance on patients to record and understand changes that were made to their medicines during remote reviews which was felt to be challenging. This is an aspect that could lead errors occurring, for example, if a patient had been identified as being at risk through the PINCER searches and changes had been recommended on the basis this, these changes may not be actioned amongst patients who are responsible for ordering their medication online. The quotation below relates to telephone consultations.

*"We [the participant and GP] will have that discussion and we will make that agreement over the telephone*. *Usually then it is where it gets a little complicated because I generally order my meds online*, *I have got to then try and remember what the doctor actually said in order to alter the medication that I have actually got in front of me …"* **(SM, Par 16)**

In recognition of challenges posed by being the recipient of a medication review consultation, suggestions were made for ways in which it could be made less burdensome for those considered more vulnerable; a population who were often referred to throughout the interview and focus groups discussions. These suggestions included allowing to individuals from a vulnerable patient’s social network to attend the review.

*“…we need to be thinking about the groups of people who cannot make the conscious decisions for themselves or even maybe contribute effectively in a review*.*”* **(PPG Focus Group 1, Par 2)** …*"Yes more vulnerable*, *that there needs to be the opportunity to have someone along with them to these reviews*…*"* **(PPG Focus Group 1, Par 7)**

However, although this was perceived to be helpful for the patient during the consultation, factors outside the consultation were considered to be potentially more difficult to manage such as coordinating appointment time with availability of the different individuals involved.

It was also advocated by participants that systems are put in place to help facilitate effective communication between health-care practitioners, vulnerable patients and those who assist them to engage in their health care activities.

*"Yes trying to find ways that enable everyone to communicate because I mean … someone mentioned elderly parents and [if] we do something where we you know we can actually intervene with that by actually speaking to someone on their behalf and you know you have to be creative about the communication systems don’t you*?.*"* **(PPG Focus Group 1, Par 9)**

#### Self-efficacy

Although there was advocacy that patient involvement is important and central to the decision making process in relation to suggested changes made on the basis of PINCER, some recognised that there are patients who are hesitant or feel less able to question the medication they are on. For these patients, it was suggested that it is the role of health care professional to initiate these types of conversations.

*“… sometimes with these things it has to be GP or pharmacist led ‘do you need it*?*’ It is very difficult for patients unless they have got a [health care] background to come in and say look should I really be on this*? *Very difficult because the patient will feel that [they are] telling the doctor what to do and then it has got to be lead from the professionals one way or another*.*"* **(PPG Group Interview 1, Par 2)**

Lacking self-efficacy was something the participants attributed to others but did not relate to or recognise in themselves. There was also some thinking that having an intervention like PINCER in place would be of particular benefit to those considered more vulnerable or less knowledgeable about their medication.

*“… I mean I am lucky in the sense that I have been on a lot of my medications for quite a while … so I have the knowledge to know oh that is not working*, *but someone … who doesn’t have that knowledge isn’t really going to know*, *oh that is a side effect of that medication*. *So … they wouldn’t really be able to pick up when a change is needed so if the GP or the pharmacist there could recognise it by the system [PINCER] then that would be beneficial*.*”* **(SM, Par 13)**

## Discussion

This is the first study to the authors’ knowledge to explore patients’ perspectives on the acceptability of the primary care prescribing safety intervention, PINCER, and propose novel insights from patient generated suggestions on how PINCER related care can be delivered in a way that is both acceptable and not unnecessarily burdensome for patients.

Overall, participants’ perceptions on the concept of PINCER were positive although there was some scepticism surrounding the possibility of it being a cost-cutting tool, which is in contrast to the perspectives of health-care professionals who reportedly consider patient safety aspects of an intervention to be more important and appealing than its cost-cutting potential [22]. A lack of understanding on the role and expertise of general practice pharmacists was identified, with those who had more knowledge on this advocating that awareness of general practice pharmacists’ role and capabilities should be raised amongst patients.

In relation to the medication review process and prescription related issues, consistency of practitioner and synchronisation of reviews and prescription renewal times were considered important. Issues surrounding the accessibility of medication review appointments were reported, and whilst delivering a medication review remotely was convenient for some, others felt it could be a barrier to effective communication between patients and health-care practitioners. Video consultations were a suggested method for remote delivery, which could help overcome communication barriers posed by telephone consultations. Although using this remote delivery method in the United Kingdom (UK) was uncommon prior to the pandemic [36], the suggestion that it could be useful was given pre-pandemic before any social distancing measures were in place or known about. Suggestions to help those considered vulnerable included allowing them to have someone else present at a review and being aware that they may be more reluctant to challenge decisions made in regard to their treatment.

Similar to our findings, a lack of awareness of the role and expertise of general practice pharmacists and the inability to differentiate between this role and that of community pharmacists has been found in other qualitative work exploring patients’ experiences of primary care [37]. Previous work has also shown that, although the scope of practice continues to grow for pharmacists in general practice [38, 39], which has been deemed beneficial by GPs and other general practice staff [40], patients do not always perceive the pharmacist’s role as being particularly important in medication counselling or monitoring [39]. Furthermore, if patients have low expectations of pharmacists, they may be less likely to be compliant to any advice given or recommended medication changes that have been initiated by a pharmacist [38]. Conversely, forming good relationships between patients and their health-care provider that foster trust, respect and effective communication has been found to lessen patients’ experience of burden, increase confidence in being able to self-manage their treatment and result in better adherence overall [41]. As it has been acknowledged that the patient-pharmacist relationship requires both parties to play an active role [39], with NICE guidelines recognising that patients with long-term conditions could benefit from pharmacists undertaking PINCER related activities [42], it further emphasises the need and importance of raising awareness of the profile and acceptability of general practice pharmacists amongst patients. This is an aspect that would seem particularly pertinent to patients who show reliance on their medical practitioner for medication advice [38]; a group who were identified as being less accepting of pharmacists and more resistant to change both in this study and in other work [38]. In a previous evaluation, patient and public representatives have suggested that GPs are in a good position to raise the profile of pharmacists amongst patients and in doing so could help foster better patient engagement and trust in pharmacist-led interventions [22]. This would therefore seem a beneficial option to explore, particularly for the aforementioned patient group who are more resistant and are less accepting of pharmacists’ involvement in care.

Participants identified medication adherence as something that PINCER would not be able to address. Although this is an aspect that was not intended to fall within the scope of PINCER, it is of relevance to medication safety [43, 44] and is therefore something that could be considered when delivering PINCER related care. In congruence with findings from this study, there has been growing recognition that patients actively seek appropriate information about their medication including associated risks or benefits, in a form that they can understand, in order to be fully involved in and comply with treatment decisions [45]. Adding to the scope of the PINCER indicators, which is something that has been suggested in a previous evaluation of a pharmacist-led intervention designed to identify patients at risk of potentially hazardous prescribing through an electronic audit and feedback surveillance dashboard [46, 47] and an unpublished PINCER process evaluation with staff and stakeholders, could be one way of addressing wider safety issues such as non-adherence whilst keeping PINCER in line current policy and priorities.

Patients living with long-term health conditions may experience less treatment burden and related implications if their care is more streamlined and their treatment regimen is comprehensible [48]. One issue raised by participants in this study was the lack of synchronisation in clinical systems used in primary care, secondary care and community pharmacies. In addition to hospital prescribed medication, over-the-counter drugs were also given as an example of medication that would not be recorded on the general practice’s clinical system even if they had been entered into the dispensing establishment’s system. It was acknowledged that these medications are therefore something that would not be picked up by PINCER and put added responsibility onto the patients to inform primary care staff that they are taking them. As the accuracy of a medication review relies on the patient being able to report being on these other medications during a consultation [49], with some patients being unaware that their GP system is not likely to store or have access to this information [49], this poses an important safety issue. One other factor worth taking into consideration in relation to streamlining of care, is inconsistency in which patients do not see the same practitioner each time they have a consultation [37, 50, 51] which was reported by some of the participants as being burdensome and a barrier towards being able to form a good working patient-practitioner relationships.

In terms of the mode in which a medication review consultation is delivered, a recent evaluation reported that patients found remote consultations to be convenient, time-efficient and less costly to attend compared to face-to-face consultations [52]. Health-care practitioners have also acknowledged that conducting consultations remotely increases the accessibility of health care appointments for patients [53]. Although the participants in this study reported similar benefits of having a remote consultation, some felt that this method could pose a barrier to effective communication in terms of being able to observe body language or pick up on non-verbal cues. Furthermore, in relation to patients considered vulnerable, an exploratory study of patients living with dementia and their carers highlighted that they experienced some difficulties with remote consultations including the lack of cues to remember points for discussion, ensuring the patient is heard, being able to deal appropriately with new and emerging issues and managing rescheduled or missed calls [54]. Of note, all remote consultations experienced by the participants in this study had been conducted via telephone but it was suggested that video consultations could help facilitate better communication. Recent work has also shown that patients and health-care practitioners find video consultations to be acceptable and provide a better platform for interaction, connectivity and communication than telephone consultations [55]. As the mode of delivery of health-care consultations has changed over the course of the pandemic and is currently more hybrid in manner, with patients reporting satisfaction with this type of service [56], tailoring PINCER related medication reviews to patient preference in terms of mode of delivery could help enhance intervention acceptability. As it has been identified that it is not always feasible or and can cause extra strain for health-care providers to offer different options for the mode of delivery [36], more work could be done to help determine how service provision could be tailored to accommodate this in a way that would not impose extra burden on patients or health-care practitioners.

In relation to the capacity of primary care, participants’ perceptions that using an intervention such as PINCER would free up more GP time and allow practitioners to focus more on their own areas of expertise were reflected in a study with health-care practitioners [57]. This cross-sectional survey highlighted that integrating a clinical pharmacist into primary care could help optimise resources, improve overall care provision, allow practitioners to dedicate time to the more fulfilling aspects of their role and reduce the risk of practitioner burnout [57].

### Strengths and weaknesses

A potential weakness, worthy of consideration, was that participants were providing opinions on PINCER based on the descriptions and explanations provided to them and their interpretations of those rather than on lived experience of being a known, direct recipient of the intervention. Due to the recruitment methods used, we were unaware, as were the participants, if PINCER had been implemented in the participants’ practices or if any of the participants had been identified through PINCER searches. However, in day to day practice, it is unlikely that a health-care practitioner would explicitly state that instigating a review or suggesting a change to a medication regimen was as a direct result of running PINCER and give in-depth details as to what the intervention entails. Furthermore, as all participants in this study were living with a long-term health condition, taking medication, had experience of interacting with primary care and had encountered medication reviews and/or monitoring, they had relevant lived experiences or knowledge of what the patient facing aspects of PINCER involve. There was also a broad age range amongst the participants and from the responses it became evident that some were living with conditions including asthma and heart failure or were taking medications such as warfarin, methotrexate, naproxen and lansoprazole, all of which are relevant to the PINCER indicators.

The focus groups and interviews commenced pre-pandemic and continued through the declaration of the pandemic and subsequent stages of restrictions. Therefore, views and opinions may have been influenced by alterations made to care delivery due to COVID-19 regulations at the time of participation, thereby posing another weakness to the study. However, the timing did allow us to capture experiences during a rapidly and continually changing landscape in care provision and patient needs making this a particular strength of the study. Furthermore, by using the TFA [23] to inform the analysis, it enabled us to gain a comprehensive, evidence-based understanding of the factors that could influence patient’s acceptability of the intervention and thereby generate suggestions on how to optimise PINCER related care from a patient’s perspective. Using this framework was particularly useful as rather than focusing on the working components of an intervention alone, it can account for views on the broader context in which an intervention has been or is intended to be delivered from a recipient or potential recipient’s perspective [23, 27].

### Implications for clinical practice and future research

On the basis of our results, it can be suggested that future research focuses on ways in which the profile of primary care pharmacists can be raised and patient-pharmacist relationships enhanced. It would also seem beneficial to establish how PINCER related care can be made more streamlined, how medication review consultations can be delivered in a feasible, flexible and acceptable way and how wider prescribing safety aspects can be addressed within the scope of PINCER. It would also seem beneficial to establish how these findings could be incorporated into current ways of working, whilst remaining in alignment with relevant policy and guidelines.

Identified in this study and, reflected in other findings [43, 49], aspects such as the use of different, unconnected clinical systems in general practice, secondary care and community pharmacists can impact upon both patient burden and medication safety. This is something that could in part be addressed by the introduction of the Discharge Medicines Service Toolkit [58], which advocates shared responsibility for medication reconciliation following discharge from hospital is taken by secondary care, primary care and community pharmacy teams. PINCER work can also be integrated into structured medication reviews (SMRs) which the NHS Long Term Plan [59] proposes that primary care network (PCN) pharmacists conduct for patients with long-term health conditions in the attempt to address medication errors. In an unpublished evaluation with staff and stakeholders, some PCN pharmacists disclosed that they were already successfully using PINCER as a tool for the purposes of their SMR work.

In relation to improving experiences of medication reviews, for example, in instances when patients felt that they were not being listened to, NICE guidelines have advocated that health-care professionals acknowledge patients’ wishes to be involved in the decision making process surrounding their medications [42]. Furthermore, any decisions made during a medication review should be done in partnership between the patient and health-care professional [42].

In terms of selecting appropriate methods for future research, conducting expert group meetings and consensus building work [60–63], which has been shown to successfully build on research findings and enhance the components and/or delivery of medicines optimisation interventions [64, 65], could be beneficial. This would include considering the points raised from the study reported in this paper in combination with findings and recommendations from previous published [16, 19–22] and unpublished evaluations with staff and stakeholders. Although these previous evaluations [16, 19–22] did have some patient and public representative input [22], they mainly focused on perspectives surrounding adoption, implementation, running PINCER and sustainable use. Overall, results from these evaluations highlighted: the importance of intervention alignment with stakeholder and policy preferences, the value taking a collaborative approach and emphasising where the intervention ‘fits’ and potentially complements other interventions [22]. Such results complement the results from this study and when combined should give a comprehensive overview of aspects that should be taken into consideration from different and relevant perspectives. Once combined recommendations can then be refined, ranked in order of importance, feasibility and acceptability for those delivering and receiving PINCER related care and decisions can be made on how they could be operationalised in practise. From this, a working model for the optimal implementation, running of the intervention and delivery of PINCER related care could be developed that is in alignment with current ways of working and relevant policies. Although in this instance, the findings will be in the context of PINCER, they could also be used to inform key components and optimal delivery of other primary care medicine optimisation based interventions.

## Conclusions

Overall, patients’ perceptions on the use of PINCER in Primary Care were positive. Delivering PINCER related care in a more streamlined manner was considered beneficial. For instance, synchronising medication reviews and prescription renewals and having consistency with the practitioner patients were consulting with were highlighted as ways that could lessen or prevent any unnecessary burden for patients. Increasing accessibility of medication review appointments and being flexible in how they are delivered, i.e. face-to-face or remotely, was also considered beneficial. Acknowledging that those considered vulnerable may need some extra assistance to engage in PINCER related care was also highlighted as a being a factor that could increase intervention acceptability and encourage patient collaboration.

## Supporting information

S1 Appendix Semi-structured interview template. (DOCX)
