# Supplementary material for: Understanding factors that could influence patient acceptability of the use of the PINCER intervention in primary care: A qualitative exploration using the Theoretical Framework of Acceptability
Source: PLoS One. 2022 Oct 14;17(10):e0275633. doi: 10.1371/journal.pone.0275633 (PMC9565699; doi:10.1371/journal.pone.0275633)
Supplement: S1 Appendix — (DOCX) [file pone.0275633.s001.docx]

S1 Appendix. Semi-structured interview template

**OPENING**

- Check if participant has read the information sheet and if they have any questions
- Complete/reiterate consent and confidentiality issues
- *We are currently carrying out a project to understand whether a service being run by pharmacists and doctors makes prescribing safer in general practice. This service involves your GP surgery using a computer programme called PINCER to help find patients who may need their medicines reviewing. Your GP practice identified you through this process and you may have had your medicines reviewed and/or changed or been called back for blood tests as a result. As part of this interview we would like to hear from you about any recent medication review and/or to your medication to discuss your thoughts and experience of the process.*

**Background information:**

1. May you tell me a little bit about yourself and whether you have had a medication review, change of medicine or blood test from your GP surgery in the last couple of years (if no, ask if ever had any of these).

**Communication regarding medication review, medication change and/or blood test**

1. If you can remember, how did you find out that a review of your medication / change of medication and/or blood test was going to be, or had, taken place? *Who informed you? How were you informed? (e.g. face-to-face, telephone, letter)*
2. If you can remember, were you told triggered this medication review/change of medication/request for blood test? *Was the reason/trigger for this discussed with you? Any mention of PINCER?*
3. ‘PINCER is like a search engine for GP computers where we can put in questions and find patients with different combinations of certain treatments and illnesses that the pharmacist and/or GP can look at in more details to see if anything needs sorting/changing or if everything is ‘OK’.’ What do you think/feel about having a tool like PINCER that searches GP computers?***.***

**Process and outcome of medication review, medicine change and/or blood tests undertaken**

1. **Medication review, medication change and/or blood tests**
   1. What opportunities did you normally have during a consultation / medicine review to ask questions and clarify information that is being presented to you?
   2. How was information usually presented to you? *(verbal vs written: leaflets, diagrams and other supportive media)? Which format did / would you prefer?*
2. What was/is the impact of the review/change/test on you? *Positive/negative impact?*
3. What was/is the impact of the review/change/test on your relationship with your doctor and/or any other staff in the practice? *Positive/negative impact?*

**PINCER awareness and role in enhancing prescribing safety**

1. How do you feel about having a pharmacist-led service such as PINCER to review your medicines? *Impact (negative or positive) on medicines being prescribed more safely?*
2. Do you think all patients should benefit from PINCER – should all practices do it? *Why do you think that?*

**CLOSING**

- Is there anything else you wish to discuss about the review and/or medicine change you recently had?
- Is there anything that you would like to go back and talk about?
- Mention what happens next: transcribing, analysis, and summarising findings to help improve PINCER roll-out
- Remind participant that if they have any questions/queries that crop up they can be in touch
- Thank you!
